# Intervertebral Disc Stem/Progenitor Cells: A Promising “Seed” for Intervertebral Disc Regeneration

**DOI:** 10.1155/2021/2130727

**Published:** 2021-07-28

**Authors:** Yuxiang Du, Zhikun Wang, Yangming Wu, Chengyi Liu, Lingli Zhang

**Affiliations:** ^1^School of Kinesiology, Shanghai University of Sport, Shanghai 200438, China; ^2^School of Physical Education & Sports Science, South China Normal University, Guangzhou, 510631 Guangdong, China

## Abstract

Intervertebral disc (IVD) degeneration is considered to be the primary reason for low back pain (LBP), which has become more prevalent from 21 century, causing an enormous economic burden for society. However, in spite of remarkable improvements in the basic research of IVD degeneration (IVDD), the effects of clinical treatments of IVDD are still leaving much to be desired. Accumulating evidence has proposed the existence of endogenous stem/progenitor cells in the IVD that possess the ability of proliferation and differentiation. However, few studies have reported the biological properties and potential application of IVD progenitor cells in detail. Even so, these stem/progenitor cells have been consumed as a promising cell source for the regeneration of damaged IVD. In this review, we will first introduce IVD, describe its physiology and stem/progenitor cell niche, and characterize IVDSPCs between homeostasis and IVD degeneration. We will then summarize recent studies on endogenous IVDSPC-based IVD regeneration and exogenous cell-based therapy for IVDD. Finally, we will discuss the potential applications and future developments of IVDSPC-based repair of IVD degeneration.

## 1. Introduction

Low back pain (LBP) is the most common musculoskeletal system disease, affecting 80% of the population. In addition to pain, LBP restricts physical activity and can lead to disabilities. Additionally, it causes a major socioeconomic burden [1–4]. Clinically, LBP always positively correlates with the degeneration of the intervertebral disc (IVD) [2, 5]. The IVD is a fibrocartilaginous tissue located between two adjacent vertebral bodies, which is a complex tissue composed of three distinct components: the nucleus pulposus (NP), the annulus fibrosus (AF), and the cartilage endplates (CEP) [6, 7]. The unique structural organization of IVD is important for spine movements, which exhibit mechanical properties such as high compressive and tensile strength and contribute to the bipedalism in humans [8, 9].

With aging, the IVD undergoes a degenerative process, known as intervertebral disc degeneration (IVDD), through cell death, extracellular matrix dehydration, and disorganization. The result of IVDD is the loss of the shock-absorbing properties of the spine [10–12]. This natural aging process can be accelerated or amplified by the accidental trauma, sedentariness, or high mechanical load [11]; however, the pathogenic mechanism of IVDD is not fully understood.

Conventional treatments of IVDD or LBP almost include conservative physical therapy, sealing therapy, and surgical treatment [13, 14]. These treatments majorly focused on relieving pain or inflammation and do not fundamentally cure the disease. Therefore, the development of new treatment strategies for IVDD is critical.

Since the 21st century, the cell-based transplantation therapy and tissue bioengineering have been used for tissue regeneration and have become a potential treatment for IVDD. In animal studies, cell transplantation therapy has been found to be effective for IVDD, using nucleus pulposus cells (NPCs), bone marrow mesenchymal stem cells (BMSCs), olfactory-derived mesenchymal stem cells (OMSCs), adipose mesenchymal stem cells (AMSCs), and induced pluripotent as cell transplantation “seeds” [15–19]. However, cell-based therapy for IVDD is limited due to the harsh environment of the intervertebral disc [19–21]. The intervertebral disc is high in osmolarity, subject to excessive mechanical loading, deficient in nutrition, low in oxygen tension, and acidic, all of which can impair the viability, proliferation, and differentiation of transplanted cells [21, 22]. Therefore, environmentally compatible cell sources are required for IVD regeneration.

Emerging evidence shows that there are endogenous stem/progenitor cells in the IVD (IVDSPCs) [23–26] that can differentiate into NPCs and reconstruct the function and construction of degenerated intervertebral discs. These properties can be an effective strategy for IVDD treatment. In this review, we will first introduce IVD, describe its physiology and stem/progenitor cell niche, and characterize IVDSPCs between homeostasis and IVD degeneration. We will then summarize recent studies on endogenous IVDSPC-based IVD regeneration and exogenous cell-based therapy for IVDD. Finally, we will discuss the potential applications and future developments of IVDSPC-based repair of IVD degeneration.

## 2. IVD Progenitor Cells in DISC Homeostasis

### 2.1. The Niche for Progenitor Cells in IVD

**Figure 1:**
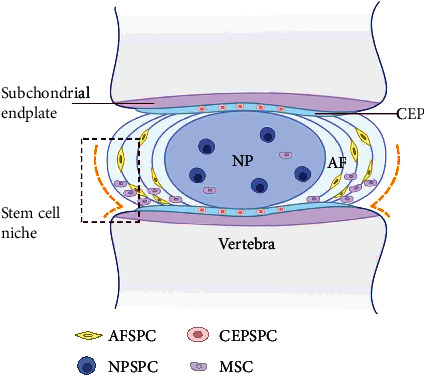
Structure of IVD and the location of IVDSPC niche. IVD is composed of three distinct regions: the gelatinous and hydrated NP at the center, the AF surrounding the NP at the periphery, and CEP above and below the IVD between adjacent vertebrae. IVDSPCs have been found in all three regions, respectively, refer to NPSPCs, AFSPCs, and CESPCs. The AF border to ligament zone and the perichondrium region adjacent to the epiphyseal plate has been suggested to be the IVDSPC niche.

The IVD is important for maintaining the stability of the spine by distributing mechanical loads and absorbing the mechanical shock produced during movement [27]. Each IVD is composed of three distinct regions that ensure a unique structure: the gelatinous and hydrated nucleus pulposus (NP) at the center of the IVD, the annulus fibrosus (AF) surrounding the NP at the IVD periphery, and cartilage endplates (CEPs) above and below the IVD between adjacent vertebrae [28]. Each region represents a structural and biological entity that participates in disc homeostasis (Figure 1).

NPs are randomly organized gelatinous tissue that contain two major types of cells: notochordal cells, which are large vacuolated cells from the embryonic notochord, and nucleopulpocytes, which are small spherical chondrocyte-like cells [11, 29, 30]. In healthy IVD, nucleopulpocytes have strong anabolic activity to synthesize abundant extracellular matrix proteins. These extracellular matrix proteins are rich in aggrecan, type II collagen, and hyaluronic acid. Aggrecan is composed of negatively charged sulfated glycosaminoglycans, making aggrecan hydrophilic. Hyaluronic acid retains water and contributes to the osmotic pressure that maintains the mechanical bearing capacity of the NPs and the vertebral disc height [31–33].

AFs are fibrocartilaginous rings composed essentially of collagen type I fibers. These fibers are radially oriented in opposite directions throughout concentric lamellae and are associated with an interlamellar matrix consisting of type II collagen, proteoglycans, and elastin [7, 28]. The AF is divided into two compartments: the outer AF, which is mainly composed of organized type I collagen fibers with high elastic and compressive properties, and the inner AF, which is a transitional area between the outer AF and the NP, which is less organized [34, 35].

CEPs interface with the adjacent vertebral bodies. CEPs are composed of hyaline cartilage cells and chondrocytes, which can synthesize abundant type II collagens and proteoglycans [11, 36]. Similar to the articular cartilage, the IVD is the most avascular tissue in the human body [28]. Although disc cells are accustomed to survive in a nutrient-deficient and hypoxia environment on the evolution, enough oxygen and glucose are necessary for disc cells for their growth and functional maintenance, which relies on the important diffusion ability of the CEPs [37, 38].

Stem cell niches are specific anatomical regions where stem cells reside in a quiescent state. Emerging evidences have been found of IVD stem/progenitor cells and stem cell niches in IVD. However, as described above, the IVD is comprised by three distinct components: the central gelatinous NP, the outer AF, and the upper and lower CEP. Thus, due to the distinct anatomical regions of IVD, the IVDSPCs are usually divided into three subsets by recent studies, respectively, nucleus pulposus-derived progenitor cells (NPSPCs), annulus fibrosus-derived progenitor cells (AFSPCs), and cartilage endplate-derived progenitor cells (CESPCs).

Risbud et al. [39] firstly identified a population of cells in the AF region of human IVD that expresses the surface markers of stem cells and have the trilineage differentiation ability (adipogenic, osteogenic, and chondrogenic differentiation). However, the detailed presence of a stem cell niche in the IVD is firstly described by Henriksson et al. [40, 41]. In their study, they detected cell proliferation zones by in vivo 5-bromo-2-deoxyuridine (BrdU) labeling in rabbits. They proposed that IVD stem cell niches might exist in the AF border to ligament zone and the perichondrium region.

Sakai et al. [23] firstly identified populations of NP progenitor cells that are Tie2 positive (Tie2+) and disialoganglioside 2 positive (GD2+) in mice and humans. They claimed that these NPSPCs can form spheroid-like colonies that express type II collagen and aggrecan. Besides, these cells could differentiate into mesenchymal lineages and induced reorganization of nucleus pulposus tissue. In the other study, Erwin et al. [42] also demonstrated that NPSPCs are present within the NP region, express genes classic for mesenchymal stem cells, and can differentiate into multiple cell lineages in vitro. In recent years, many studies have reported the existence of NPSPCs in various animal models; these cells are believed to have the ability to contribute to the cellular organization in the IVD.

Furthermore, the results from Kim et al. illustrated that rabbit notochordal cells could stimulate the migration of cells from the CEP into the NP region and changed a notochordal NP into a fibrocartilaginous NP [43, 44]. In 2011, Liu et al. successfully isolated a universal cell population with stem cell characteristics from human CEPs, which are superior in terms of the osteogenic and chondrogenic capacity in vitro [45]. Besides, Xiong et al. [46] also isolated and identified a population of CEPSCs, which could migrate from CEP to NP tissue, and this migration could be inhibited by macrophage migration inhibitory factor.

The discovery of CEPSCs is important for the future of IVDD cell-based therapy. Current cell-based therapies mainly target NP and AF degeneration, but calcification of the CEP can affect IVD regeneration. CEP calcification can block nutrient supply to the endogenous or implanted cells and impair the cell regeneration process.

Recently, some researchers proposed that the stem cell niche in the IVD is a dynamic microenvironment consisting of the unique extracellular cell matrix (ECM) and neighboring cells that can regulate local stem/progenitor cells [41, 47]. In other words, despite the different locations of stem/progenitor cells in the different regions in IVD, the AFSPCs, NPSPCs, and CESPCs may contain the same population of stem/progenitor cells which have the same origin and biological characteristics. In brief, from these studies, we can definite the existence of endogenous IVDSPCs in different regions both in an animal model and in human IVD.

### 2.2. Properties of IVD Progenitor Cells

Previous studies have already showed that IVDSPCs have many similar surface markers with marrow mesenchymal stem cells (MSCs) [48], which express CD73, CD90, and CD105 but not CD34, CD45, CD14, CD11b, CD79, CD19, and HLA-DR. The current characterization and related study findings of IVD progenitor cell characteristics are described in Table 1. Besides, IVDSPCs may also express markers known to be expressed by other types of stem/progenitor cells. For instance, clonal cells isolated from nonchondrodystrophic canine NP cells expressed the pluripotent stem cell marker genes SOX2, OCT3/4, and NANOG [42, 49]. According to the studies, a small proportion of human IVDSPCs also express CD133 [39, 50], a surface glycoprotein usually expressed on many types of stem cells and cancer stem cells [51, 52]. A minor population of human NP cells from degenerated IVDs also express CD34 [53], which is a marker for haematopoietic stem cells and some progenitor cells. Notably, previous studies have shown that IVD cells, such as NPs and AFs, also express surface markers similar to mesenchymal stem cells, making it harder to distinguish the IVDSPCs from other IVD cells [23]. Therefore, it is necessary to explore the distinct surface markers of IVDSPCs. For instance, Sakai and his group [23] have also indicated that the tyrosine kinase endothelial receptor (Tie2) and disialoganglioside 2 (GD2) are sensitive markers of NPSPCs. Besides, with the development of single-cell sequencing technology, it has already been a powerful tool to identify new clusters of stem/progenitor cells in other tissues, which can help to clearly classify subpopulations of all IVD cells.

In addition to surface marker expression, MSCs also should be plastic adherent and be able to undergo osteogenesis, adipogenesis, and chondrogenesis, as defined by the International Society for Cellular Therapy. In this respect, IVDSPCs also have compared properties with MSCs. For example, CESPCs are reported to have a similar proliferation rate but a better osteogenic and chondrogenic ability compared to BMSCs [45]. In a rat model, Zhang et al. [54] reported that NPSPCs have a similar proliferative capacity with BMSCs. However, Wu et al. [55] found that NPSPCs were expressed less than MSC surface markers and exhibited reduced proliferation capability and differentiation potentials than umbilical cord-derived MSCs [55]. What is more, in the continuous studies of Chen et al. [56, 57], they reported that NPSPCs have a greater viability, proliferation rate, and ECM metabolism than AMSCs under hypoxic conditions or acidic conditions. By the way, many studies have showed that hypoxic conditions promote differentiation in MSCs into NP-like cells [58–60]. Besides, Liang et al. [61] compared the biological characteristics of NPSPCs, AFSPCs, and CESPCs from human IVD. They concluded that the cell morphology of these three kinds of IVDSPCs was not significantly different. However, in vitro proliferation and differentiation assay demonstrated that AFSPCs had the best stem-cell-like characteristics. However, Wang et al. [62] reported that there was no significant difference in proliferation ability among NPSPCs, AFSPCs, and CESPCs and claimed that CESPCs have the strongest osteogenic and chondrogenic potentials compared to NPSPCs and AFSPCs.

Although intervertebral discs have been characterized, these studies are predominantly in vitro, which may be different in vivo. Discrepancies that exist between studies may be explained in part by the different species or cell isolation and culturing protocols. Thus, future studies should focus on identifying IVDSPC properties in vivo by fluorescent-labeled animal models and explore the localization of the IVDSPCs in the IVD development by lineage tracing or fate mapping.

## 3. IVD Progenitor Cells in DISC Degeneration

IVDD is a progressive disease caused by genetics, aging, and injuries that leads to molecular, cellular, structural, and mechanical changes of the IVD [63–66]. As degeneration occurs, loss of proteoglycans and hydration in NP affects homeostasis in the ECM, mechanical properties, and shock absorber function. As such, mechanical force should be distributed evenly over the IVD because it can increase in the tensile forces exerted on the AF and accelerate degeneration. A protruding NP would crush the AF, leading to the compression of the adjacent sensory nerve and subsequently cause LBP [67, 68]. As IVDD progresses, increased secretion of inflammatory factors and other catabolic factors promote blood vessels and sensory nerves to grow into NP and AF region and amplify pain through a degenerative cascade [63, 67]. The IVD relies on CEP to exchange nutrients and metabolites. However, the CEP is gradually calcified during IVDD. This calcification impedes nutrient diffusion and metabolite releases, resulting in an acidic and nutritionally deficient environment for IVDSPCs [11, 69].

**Figure 2:**
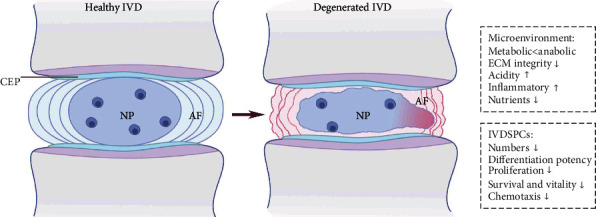
Changes of the IVD microenvironment and IVDPCs in normal IVD versus degeneration. IVDD can lead to decreased metabolism and increased anabolism in NP cells, resulting in loss of proteoglycans and hydration in NP, as well as bad impacts on the ECM integrity. The degenerated IVDs are usually acidic, inflammatory, and nutritionally deficient, which provide an adverse microenvironment for IVDSPCs. IVDD can result in decreased chemotaxis, survival, and vitality of the IVDSPCs, leading to the decreased numbers of IVDSPCs. IVDSPCs in degenerated discs also exhibit impaired differentiation potency and proliferation capacity.

Similar to many endogenous progenitor cells, IVDSPCs can also maintain a dynamic balance between healthy IVD and degenerated IVD [22, 24]. Several studies have reported the changes of IVDSPC as IVD degenerated (Figure 2). Hypoxia, acidic, high mechanical loading, and inflammatory environment in degenerative IVD can result in reduced number and migration ability of IVDSPCs. For example, hypoxia can inhibit the viability and proliferation of NPSPCs but promote the chondrocytic differentiation of NPSPCs^56^. Mizrahi et al. [70] compared NPSPC function from healthy and degenerated porcine discs and found that NP cells from degenerate discs have superior proliferation ability in vitro. Liu et al. [71] reported that acidic conditions could significantly inhibit cell proliferation and increase cell apoptosis of IVDSPCs. The acidic microenvironment in degenerated IVD can also inhibit the viability and proliferation ability of the IVDSPCs, as well as the reduction of aggrecan, collagen II, and metalloproteinase-3 and the increase of matrix metalloproteinase-2 and thrombospondin motifs-4 expression in NPSPCs^57^. Besides, as degeneration occurred, the increased mechanical stress including compression loading and various kinds of stress may significantly affect IVDSPCs [72, 73]. For example, Deng et al. [74] reported that continuous stress could affect endogenous IVDSPC migration. Liang et al. [75] also found that the increased compression loading could inhibit the viability, differentiation, colony formation, and migration ability of IVDSPCs and downregulate the expression of stem cell-related genes. What is more, inflammation in degenerated IVD can also impact IVDSPCs [76, 77] by increasing IL-1*β* and inhibiting the proliferation and anabolism of IVDSPCs [78]. Overall, these studies show the degenerated IVD microenvironment affects IVDSPCs; thus, improving the microenvironment to activate endogenous IVDSPCs is promising for IVDD.

## 4. IVD Progenitor Cells in DISC Regeneration

### 4.1. Traditional Cell-Based Therapy

Current treatments do not target the fundamental problems of IVDD, such as the degenerated disc dysfunction and structure disorder structure. With the growing interest in cell transplantation for tissue regeneration and a better understanding of the IVDD pathophysiology, cell-based therapy is a potential treatment for IVDD. Cell-based therapies are expected to restore the biomechanical functions of the IVD, as well as the acidic, hypoxic, and nutrient-deficient microenvironment. Therefore, it is imperative that the cell “seed” for cell-based therapy should have the strong ability of survival, proliferation, and differentiation and the ability to rebuild a comparable microenvironment of the IVD.

In the early 21st century, MSCs were one of the most used cell populations for regeneration. MSCs, such as BMSCs, OMSCs, and AMSCs, can differentiate into NP-like cells in vitro [79, 80] and restore radiographically assessed IVD height in animal models, which might be an effective strategy for the treatment of IVDD.

BMSCs and AMSCs are commonly used as cell-transplant seeds due to their accessibility and excellent differentiation abilities and have been found to be effective for improving degenerated IVD. Hussain et al. [81] delivered BMSC-seeded high-density collagen into injured IVDs of sheep. Within six weeks, the disc high index improved, which indicated a good repair of the IVD. Noriega et al. [82] transplanted BMSCs into degenerated IVDs of LBP patients by intradiscal injection. Clinical outcomes were followed for 1 year and included evaluation of pain, disability, and quality of life. The BMSC-treated patients displayed quick and significant improvement in algofunctional indices compared to the healthy controls. Wang et al. [16] preconditioned BMSCs in hypoxia before transplantation and saw enhanced cell survival in degenerated rat IVD. Elabd et al. [83] also demonstrated clinical safety and feasibility of the clinical use of hypoxic pretreated BMSCs for the treatment of IVDD in five patients. Magnetic resonance imaging (MRI) showed no abnormalities surrounding the treated region, and all patients self-reported overall improvement of LBP. Thus, pretreated MSCs before transplantation provided a new way to improve the effects of BMSC-based transplantation in IVDD. Moreover, Ishiguro et al. [84] developed an AMSC-based scaffold-free tissue-engineered construct as a novel cell therapy system for IVDD. The AMSC-based implantation system restored the IVD integrity and the biomechanical function in a rat tail model without destruction of surrounding vertebral structures. Recently, Muttigi et al. [85] optimized the matrilin-3 priming dose, duration, and culture conditions for the preparation of AMSC spheroids. They found that this system could enhance the secretion of growth factors to induce repair and restored and rehydrated the IVD microenvironment.

Besides, induced pluripotent stem cells (iPSCs) are another good choice for cell-based therapy for IVDD. Setton et al. [86, 87] promote iPSC differentiation into NP-like cells in vitro and provide a cell source for the development of new therapies for IVD diseases. However, they do not demonstrate the treatment efficacy of iPSC-derived NPs for IVD regeneration in animal models. Recently, Zhang et al. [88] reported that notochord-like and NP-like cells can be derived from human pluripotent stem cells using a compound-defined protocol. These iPSC-derived NP-like cells share high similarities with adolescent NP cells and attenuate injury-induced intervertebral disc degeneration after transplantation.

### 4.2. IVDSPC-Based IVD Endogenous Regeneration

Although exogenous MSC-based therapy is a promising treatment for IVDD, however, successful implementation of exogenous cell-based therapy is challenged by the specialized microenvironment of the IVD. Even in the steady state, the IVD is avascular, hypoxic, nutritionally deficient, and high biomechanical loading, which is hard to survive for exogenous cells. Unfortunately, all of these bad conditions would be exacerbated when IVDD occurred as described above, which seriously hindered the application of exogenous stem/progenitor cell-based treatment for IVDD. Furthermore, due to the uncontrollability of proliferation and differentiation of exogenous stem/progenitor cells in vivo, their clinical utility is also hampered by their osteogenic and tumorigenic potential [89]. Therefore, environmentally compatible cell sources, such IVDSPCs, are required.

When tissue is damaged, the inflammatory cells would quickly move to the injury site and release various kinds of cytokines to recruit endogenous stem/progenitor cells. Then, the recruited endogenous stem/progenitor cells undergo proliferation and differentiation to begin tissue regeneration. In recent years, many studies confirmed that endogenous stem/progenitor cells are an important source for tissue self-regeneration in many tissues, especially in the musculoskeletal system. For example, Lee et al. [90] identified a rare population of CD146+ tendon progenitor cells, which exhibited clonogenic capacity and multilineage differentiation ability. They also demonstrated that the CD146+ tendon progenitor cells could contribute to the regeneration after tendon injury. Using a biodegradable material combined with Gli1+ endogenous suture MSCs, Yu et al. [91] successfully regenerated a functional cranial suture that corrects skull deformity, normalizes intracranial pressure, and rescues neurocognitive behavior deficits in Twist1+/− mice with craniosynostosis. All these studies indicate the great potential of endogenous stem/progenitor cells for injury regeneration in the skeletal system.

While endogenous repair is also present in the IVD^22^, injury to the IVD affects the repair process by stem/progenitor cells. The reason for the failure of IVD endogenous repair is likely due to the insufficient endogenous progenitor cell recruitment to the damaged site and the adverse IVD microenvironment affecting the activity and viability of IVD progenitor cells [22]. Therefore, recruiting enough IVDSPCs and reforming the adverse microenvironment in the degenerated IVD must be a primary strategy for the endogenous repair of IVDD. For instance, Pattappa et al. [92] reported that CCL5 is a key chemoattractant that is produced and released by the intervertebral disc cells. Therefore, these factors enhance stem/progenitor cell mobilization in regenerative therapies for early stages of disc degeneration. Ying et al. [93] also found that stromal cell-derived factor-1*α* (SDF-1*α*) released in the degenerated IVD can activate and recruit endogenous NPSPCs. They suggested that SDF-1*α* is important for in situ IVDD regeneration.

Utilizing chemokines to recruit endogenous or exogenous progenitor cells is a promising strategy for improving endogenous IVD regeneration, especially when combined with compatible biomaterials. Pereira et al. [94] used hyaluronan-poly(N-isopropylacrylamide) hydrogel as a chemoattractant delivery system to slow-release SDF-1 when transplanted into the degenerated IVD. This system could enhance both the number of recruited IVDSPCs and their migration distance in the IVD. Notably, Frapin and colleagues [95] developed a new delivery system composed of pullulan microbeads that deliver CCL5 to promote recruitment of IVDSPCs, as well as TGF-*β*1 and GDF-5 synergistic cocktail to stimulate the synthesis of an NP-like ECM synthesis. This strategy promoted endogenous repair of IVD. In another respect, the degenerated IVD environment is hypoxic, acidic, inflammatory, and high mechanical loading as described above, which results in reduced mobilization of IVDSPCs. Therefore, overcoming the adverse microenvironment in the IVD to promote endogenous repair is a promising strategy as well. For example, Liu et al. [96] reported that the antioxidative and anti-inflammatory properties of aspirin significantly improve IVDD. Zhang et al. [97] also demonstrated that melatonin could modulate ECM remodeling and attenuate the IVDD in a rat tail puncture model by resisting the inflammatory cytokines. Using inhibiting an acid-sensing ion channel can reduce the adverse effects of an acidic environment on NPSPCs by allowing an extracellular receptor to maintain function in an acidic environment [71]. These studies demonstrate that improving the microenvironment for cell-based therapy can improve outcomes.

### 4.3. Future IVDSPC-Based Therapy for IVD Regeneration

Despite the advantages of IVDSPCs for disc regeneration, there are some limitations to the clinical application of IVDSPC-based transplantation therapy for IVD regeneration. First, current understanding of IVDSPCs is insufficient, hampering the discovery of new IVDSPCs and even the development of IVDSPC-based therapy for IVDD. As single-cell sequencing technology develops, we need to understand the role of heterogeneous cell populations in the NPs, AFs, and even CEPs for IVDSPCs. Wang et al. [98] used the Sc-RNA sequencing technology to divide the disc cells into five categories at the single-cell level. Among the five groups, they identified a population of cells (Eng+ and Mcam+) with a stem cell-like phenotype by tracking common MSC marker genes. Although they did not demonstrate the proliferation and differentiation ability of these cells, this study paved the way for further research on the clarification of subpopulations of disc cells by Sc-RNA sequencing technology, which could further contribute to the discovery of a powerful cell “seed” for cell-based therapy for IVDD.

Second, more appropriate and compatible materials need to be designed to serve as a more suitable carrier for IVDSPCs. Compared to the normal liquid carrier, biomaterials can avoid the cell leakage from the pinhole of the intervertebral space and provide a suitable environment for cell “seeds.” These properties can enhance the curative effect of cell-based therapy. There are mainly two types of biomaterials for intervertebral disk regeneration: biomaterials with high mechanical strength and biomaterials with certain elasticity and softness. Isa and collegues [99] found that HA hydrogel alleviated the pain in a rat disc injury model. This study suggests that HA hydrogel may have an injectable carrier for IVDSPCs that could be translated to degenerated disc. Chen et al. [100] developed a multifunctional polyethylene glycol hydrogel with injectable and self-healing properties. They also loaded a small molecule drug, Agomir874, into this system to mimic miRNA874 by downregulating the expression of matrix metalloproteinases in the NP and restoring the extracellular microenvironment. To some extent, some growth factors that benefit for IVDSPCs' proliferation or vitality could be added in the biomaterial for disc regeneration ref. Despite the great potential of biomaterials for cell therapy, some problems remained. Sometimes, these materials may induce CEP damage because of their high mechanical strength. Contrastingly, biomaterials that are too soft can break into fragments because of the high mechanical load of IVD. Therefore, biomaterials with appropriate elasticity and stiffness should be developed for delivering IVDSPCs into the IVD and growth factors could be used to improve the microenvironment for transplanted IVDSPCs.

Finally, although cell-based therapy might be an effective approach for the treatment of IVDD, many obstacles remained [21, 89]. For example, Gianluca et al. [89] reported that transplanted cells could form osteophyte and might migrate out of the IVD. The uncontrollable proliferation of transplanted cells also hampered the clinical utility of cell-based therapy because of tumorigenic potential [89, 101]. What is more, the cell-based therapy for IVDD requires a large number of injected cells (usually ~10 [7]) [20]. Although IVDSPCs can perform as a good cell resource for the cell therapy of IVDD, the difficult collection and insufficient number of endogenous IVDSPCs may hamper the clinical utility of IVDSPCs. Therefore, there has been a shift toward cell-free therapies for IVDD to circumvent the aforementioned obstacles [102–104]. Extracellular vesicles (EVs) are lipid membrane particles carrying proteins, lipids, DNA, and various types of RNA that are involved in intercellular communication. EVs derived from stem/progenitor cells have been suggested to be excellent therapeutic agents in many diseases, including IVDD [105–108]. Many different groups have demonstrated that MSC-derived EVs can inhibit apoptosis and promote the proliferation of the IVD cells. For example, Cheng et al. [109] reported that BMSC-derived EVs can inhibit NP cell apoptosis and reduce IVDD through miR-21. Xiang et al. [110] and Liao et al. [111], respectively, demonstrated that MSC-derived EVs can attenuate ER stress-induced NP cell apoptosis and retarded IDD progression in a rat model. Besides, Hingert et al. [112] treated human degenerated disc cells with MSC-derived EVs in vitro and saw more than a 50% increase in cell proliferation and a decrease in apoptosis. Recently, Guo et al. [113] reported that MSC-derived EVs can promote NP cell proliferation. They found that matrilin-3, an extracellular matrix protein, is enriched in EVs and is necessary for EVs to promote the proliferation of NP cells. In addition to affecting cell proliferation and apoptosis, EVs can also play an important role in metabolic programming of disc cells. For an instance, Li et al. [114] found that MSC-derived EVs can reduce ECM degradation by downregulating matrix-degrading enzymes and promote the anabolism of collagen II and aggrecan, protecting NP cells from acidic pH-induced apoptosis. Guo et al. [113] also found that MSC-derived EVs can promote ECM synthesis of NP cells. These studies suggested that MSC-derived EVs exert a protective effect on disc cells by attenuating the pathological environment in IVD. However, there are few reports about the influences by using IVDSPC-derived EVs to treat degenerated disc cells. In view of the better biocompatibility of IVDSPCs in the harsh IVD environment than exogenous MSCs, IVDSPC-derived EVs may have an excellent effect in protecting disc cells from degeneration and contribute to the regeneration of the IVD.

## 5. Conclusion

IVDD is a primary reason for LBP, causing an enormous economic and medical burden due to the inadequate efficacy of traditional clinical therapy. The discovery of IVD endogenous stem/progenitor cells brings new insight for IVDD treatment, which might be an excellent cell “seed” for IVD regeneration. The IVDSPCs are like MSCs in surface markers, proliferation, and differentiation, with the added benefit of being able to withstand the harsh microenvironment of degenerated IVD. These properties allow IVDSPCs to successfully IVD regeneration. Because IVDSPCs can be extracted from patients with disc herniation avoiding additional damage, the IVDSPCs are more accessible than other types of stem/progenitor cells.

However, few studies have reported the biological properties of the IVDSPCs in detail and their potential application, limiting our knowledge about these cells. In particular, the current understanding of IVDSPCs is mainly based on in vitro experiments, which may differ in vivo. IVDSPC properties are still controversial depending on the type of cell (NPSPCs, AFSPCs, and CEPSPCs). Therefore, further studies should focus on the accurate identification of the IVDSPC properties in vivo based on new technologies and animal models. Preclinical studies are required for improving the therapeutic effect of the IVDSPCs for clinical translation. Future studies should also focus on improving the endogenous repair of IVD based on endogenous IVDSPCs to promote IVDSPC migration to damaged IVD tissues and improve the activity and viability of the IVDSPCs. More compatible biomaterials should be developed to load the IVDSPCs into the degenerated IVD for cell-based transplantation. Because cell-free therapies can circumvent obstacles caused by traditional cell-based therapies, the IVDSPC-derived factors, such as EVs, are also powerful therapeutic agents for IVDD. Overall, IVDSPCs are a promising cell “seed” for IVD regeneration.

## Figures and Tables

**Table 1:** Identification and surface markers of IVDSPCs.

Species	Cell type	Surface markers	References
Human	NPSPCs	CD73+, CD90+, CD105+, CD29-CD45-	Qi et al., 2019 [115]
Human	NPSPCs	CD73+, CD90+, CD105+, CD34-, CD45-, HLA-DR-	Liu et al., 2017 [71]
Human	NPSPCs	CD73+, CD90+, CD105+, CD34-, CD45-	Chen et al., 2016 [116]
Human	NPSPCs	Tie2+, GD2+, Flt1+, CD271+, CD24-	Sakai et al., 2012 [23]
Human	NPSPCs	CD90+, CD73+, CD105+, CD106+, CD166+, CD14-, CD19-, CD24-, CD34-, CD45-, HLA-DR-	Blanco et al., 2010 [117]
Human	NPSPCs	CD29+, CD44+, CD105+, CD14-, CD34-, CD45-, HLA-DR-	Shen et al., 2015 [25]
Human	NPSPCs	Tie2+, GD2+, Nanog+, Oct-4+, Sox-2+	Li et al., 2017 [118]
Rat	NPSPCs	CD73+, CD90+, CD105+, CD34-, CD45-	Zhao et al., 2017 [119]
Canine	NPSPCs	CD133+, KI67+, Nanog+, NCAM+, Nestin+, Oct3/4+, Sox2+, Brachyury-	Erwin et al., 2013 [42]
Human	NPSPCs, AFSPCs	CD49a+, CD63+, CD73+, CD90+, CD105+, CD166+, p75 NTR+, CD133/1+, CD34-	Risbud et al., 2007 [39]
Rhesus macaque	NPSPCs, AFSPCs	CD44+, CD90+, CD146+, CD166+, HLA-DR+, CD271-, CD106-, CD29-	Huang et al., 2013 [120]
Rabbit	NPSPCs, AFSPCs	PCNA+, CD166+, C-kit+, Jagged1+, Notch1+	Yasen et al., 2013 [121]
Human, rat, rabbit	AFSPCs	Notch1+, Delta4+, CD117+, STRO-1+, C-kit low, KI67 low	Henriksson et al., 2009 [40] Henriksson et al., 2012 [41]
Human	AFSPCs	CD29+, CD49e+, CD51+, CD73+, CD90+, CD105+, CD166+, CD184+, CD24+, Stro-1+, Nestin+, NSE+, CD31-, CD34-, CD45-, CD106-, CD117-, CD133-	Feng et al., 2010 [122]
Human	AFSPCs	CD14+, CD29+, CD44+, CD73+, CD90+, CD105+, STRO-1+, CD34-, CD45-	Gruber et al., 2016 [123]
Rabbit	AFSPCs	CD29+, CD44+, CD166+, Oct4+, Nucleostemin+, SSEA-4+, CD4-, CD8-, CD14-	Guo et al., 2018 [124]
Rabbit	AFSPCs	CD29+, CD44+, CD166+, Oct4+, SSEA-4+, CD4-, CD8-, CD14-	Liu et al., 2014 [125]
Human	CEPSCs	CD73+, CD90+, CD105+, CD34-, CD45-, HLA-DR-	Yuan et al., 2018 [126] Yao et al., 2016 [127]
Human	CEPSCs	CD44+, CD73+, CD90+, CD105+, CD133+, CD166+, Stro-1+, CD14-, CD19-, CD34-, CD45-, HLA-DR-	Liu et al., 2011 [45] Huang et al., 2012 [50]
